# 10-Hydr­oxy-2-aza­penta­cyclo­[10.8.0.0^2,10^.0^4,9^.0^15,20^]icosa-1(12),4(9),5,7,13,15(20),16,18-octa­ene-3,11-dione

**DOI:** 10.1107/S1600536811005356

**Published:** 2011-02-23

**Authors:** Saeedeh Hashemian, Behrouz Notash

**Affiliations:** a Department of Chemistry, Islamic Azad University, Yazd Branch, Yazd, Iran; b Department of Chemistry, Shahid Beheshti University, G. C., Evin, Tehran 1983963113, Iran

## Abstract

In the title compound, C_19_H_11_NO_3_, the isoindolinone ring system is approximately planar with a maximum atomic deviation of 0.071 (1) Å and the five-membered ring of the dihydro­benzo[*g*]indol-3-one unit assumes an envelope conformation. The naphthalene ring system makes a dihedral angle of 39.47 (4)° with the mean plane of the isoindolinone system. Inter­molecular O—H⋯O and C—H⋯O hydrogen bonding helps to stabilize the crystal structure.

## Related literature

For applications of naphthyl­amines, see Valenti *et al.* (2006 ▶); Black *et al.* (1994 ▶).
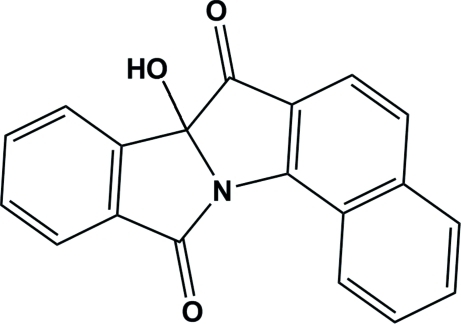


## Experimental

### 

#### Crystal data

C_19_H_11_NO_3_
*M* *_r_* = 301.29
Triclinic, 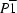

*a* = 7.3250 (11) Å
*b* = 9.7916 (16) Å
*c* = 10.4532 (17) Å
α = 70.401 (13)°
β = 82.503 (13)°
γ = 75.862 (12)°
*V* = 683.94 (19) Å^3^
*Z* = 2
Mo *K*α radiation
μ = 0.10 mm^−1^
*T* = 298 K
0.40 × 0.30 × 0.29 mm

#### Data collection

Stoe IPDS II diffractometer
7737 measured reflections
3627 independent reflections
3161 reflections with *I* > 2σ(*I*)
*R*_int_ = 0.040

#### Refinement

*R*[*F* ^2^ > 2σ(*F* ^2^)] = 0.044
*wR*(*F* ^2^) = 0.136
*S* = 1.13
3627 reflections
212 parameters
H atoms treated by a mixture of independent and constrained refinement
Δρ_max_ = 0.29 e Å^−3^
Δρ_min_ = −0.22 e Å^−3^

### 

Data collection: *X-RED32* (Stoe & Cie, 2005 ▶); cell refinement: *X-AREA* (Stoe & Cie, 2005 ▶); data reduction: *X-AREA*; program(s) used to solve structure: *SHELXS97* (Sheldrick, 2008 ▶); program(s) used to refine structure: *SHELXL97* (Sheldrick, 2008 ▶); molecular graphics: *ORTEP-3 for Windows* (Farrugia, 1997 ▶); software used to prepare material for publication: *WinGX* (Farrugia, 1999 ▶).

## Supplementary Material

Crystal structure: contains datablocks I, global. DOI: 10.1107/S1600536811005356/xu5159sup1.cif

Structure factors: contains datablocks I. DOI: 10.1107/S1600536811005356/xu5159Isup2.hkl

Additional supplementary materials:  crystallographic information; 3D view; checkCIF report

## Figures and Tables

**Table 1:** Hydrogen-bond geometry (Å, °)

*D*—H⋯*A*	*D*—H	H⋯*A*	*D*⋯*A*	*D*—H⋯*A*
O2—H2⋯O1^i^	0.96 (2)	1.85 (2)	2.7978 (15)	172.2 (17)
C6—H6⋯O3^ii^	0.93	2.52	3.1609 (18)	126

Symmetry codes: (i) 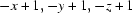; (ii) 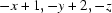.

**Fig. 1.**
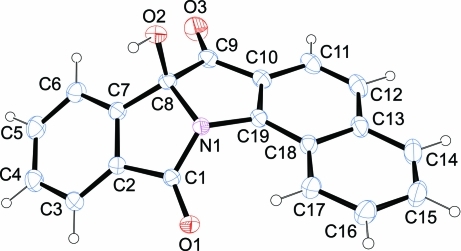
The molecular structure of the title compound with displacement ellipsoids drawn at 30% probability level.

**Fig. 2.**
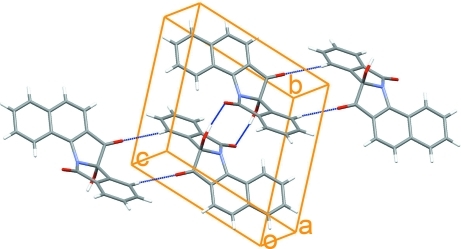
The packing diagram of the title compound. The intermolecular O—H···O and C—H···O hydrogen bonds are shown as blue dashed lines.

## supplementary crystallographic information

### Comment

Naphthylamine is an aromatic amine which can be obtained from nitronaphthalene. Naphthylamines are used in the manufacture of dyes, condensation colors, and rubber and in the synthesis of a large number of chemical (Black *et al.* 1994; Valenti *et al.* 2006). Ninhydrin (2,2-dihydroxyindane-1,3-dione) is a chemical used to detect ammonia or primary and secondary amines. The carbon atom of a carbonyl bears a partial positive charge enhanced by neighboring electron withdrawing groups like carbonyl itself. So the central carbon of a 1,2,3-tricarbonyl compound is much more electrophilic than one in a simple ketone. Thus indane-1,2,3-trione reacts readily with nucleophiles, including water. Whereas for most carbonyl compounds, a carbonyl form is more stable than a product of water addition (hydrate), ninhydrin forms a stable hydrate of the central carbon because of the destabilizing effect of the adjacent carbonyl groups.

We report here the crystal structure of 7-hydroxy-7-aH-benzo[*g*] isoindolino[1,2-α]indole-7,10-dione. The title compound was prepared by the reaction of α-naphthylamine and ninhydrine in molar ratio of 1: 1 in acetone. The structure of title complex is shown in Fig. 1. There are intermolecular hydrogen bondings between O—H···O and C—H···O which play important role in the stabilization of crystalline network (Table 1 & Fig. 2).

### Experimental

The title compound was prepared by the reaction of α- naphthylamine and ninhydrine in molar ratio of 1: 1 in acetone. The mixture was stirred for about 2 h at room temperature. The resulting solution was kept in air. After slow evaporation, single crystals of the title compound suitable for an X-ray diffraction study formed at the bottom of the vessel after 2 weeks (m.p. 138 °C).

### Refinement

Hydroxyl H atom was found in a difference Fourier map and refined isotropically. Other H atoms were positioned geometrically and refined as riding atoms with C—H = 0.93 Å and *U*_iso_(H) = 1.2*U*_eq_(C).

### Crystal data

**Table:**

C_19_H_11_NO_3_	*Z* = 2
*M**_r_* = 301.29	*F*(000) = 312
Triclinic, *P*1	*D*_x_ = 1.463 Mg m^−^^3^
Hall symbol: -P 1	Mo *K*α radiation, λ = 0.71073 Å
*a* = 7.3250 (11) Å	Cell parameters from 1011 reflections
*b* = 9.7916 (16) Å	θ = 2.3–25.0°
*c* = 10.4532 (17) Å	µ = 0.10 mm^−^^1^
α = 70.401 (13)°	*T* = 298 K
β = 82.503 (13)°	Block, colorless
γ = 75.862 (12)°	0.40 × 0.30 × 0.29 mm
*V* = 683.94 (19) Å^3^	

### Data collection

**Table:**

Stoe IPDS II diffractometer	3161 reflections with *I* > 2σ(*I*)
Radiation source: fine-focus sealed tube	*R*_int_ = 0.040
graphite	θ_max_ = 29.3°, θ_min_ = 2.3°
rotation method scans	*h* = −10→10
7737 measured reflections	*k* = −13→13
3627 independent reflections	*l* = −14→14

### Refinement

**Table:**

Refinement on *F*^2^	Primary atom site location: structure-invariant direct methods
Least-squares matrix: full	Secondary atom site location: difference Fourier map
*R*[*F*^2^ > 2σ(*F*^2^)] = 0.044	Hydrogen site location: inferred from neighbouring sites
*wR*(*F*^2^) = 0.136	H atoms treated by a mixture of independent and constrained refinement
*S* = 1.13	*w* = 1/[σ^2^(*F*_o_^2^) + (0.072*P*)^2^ + 0.1037*P*] where *P* = (*F*_o_^2^ + 2*F*_c_^2^)/3
3627 reflections	(Δ/σ)_max_ < 0.001
212 parameters	Δρ_max_ = 0.29 e Å^−^^3^
0 restraints	Δρ_min_ = −0.22 e Å^−^^3^

### Special details

**Table:**

Geometry. All e.s.d.'s (except the e.s.d. in the dihedral angle between two l.s. planes) are estimated using the full covariance matrix. The cell e.s.d.'s are taken into account individually in the estimation of e.s.d.'s in distances, angles and torsion angles; correlations between e.s.d.'s in cell parameters are only used when they are defined by crystal symmetry. An approximate (isotropic) treatment of cell e.s.d.'s is used for estimating e.s.d.'s involving l.s. planes.
Refinement. Refinement of *F*^2^ against ALL reflections. The weighted *R*-factor *wR* and goodness of fit *S* are based on *F*^2^, conventional *R*-factors *R* are based on *F*, with *F* set to zero for negative *F*^2^. The threshold expression of *F*^2^ > σ(*F*^2^) is used only for calculating *R*-factors(gt) *etc*. and is not relevant to the choice of reflections for refinement. *R*-factors based on *F*^2^ are statistically about twice as large as those based on *F*, and *R*- factors based on ALL data will be even larger.

### Fractional atomic coordinates and isotropic or equivalent isotropic displacement parameters (Å^2^)

**Table:**

	*x*	*y*	*z*	*U*_iso_*/*U*_eq_	
C1	0.60706 (15)	0.62937 (12)	0.49240 (11)	0.0298 (2)	
C2	0.69780 (16)	0.61664 (13)	0.36032 (12)	0.0324 (2)	
C3	0.87442 (18)	0.53663 (16)	0.33404 (14)	0.0410 (3)	
H3	0.9488	0.4711	0.4042	0.049*	
C4	0.9358 (2)	0.55825 (19)	0.19890 (16)	0.0494 (3)	
H4	1.0540	0.5065	0.1779	0.059*	
C5	0.8237 (2)	0.65604 (19)	0.09393 (15)	0.0502 (3)	
H5	0.8694	0.6697	0.0042	0.060*	
C6	0.6452 (2)	0.73333 (16)	0.12066 (13)	0.0425 (3)	
H6	0.5690	0.7968	0.0507	0.051*	
C7	0.58481 (16)	0.71222 (13)	0.25611 (12)	0.0332 (2)	
C8	0.40104 (16)	0.77784 (12)	0.31604 (11)	0.0318 (2)	
C9	0.33814 (17)	0.94765 (13)	0.28511 (13)	0.0374 (3)	
C10	0.29102 (17)	0.97079 (13)	0.41761 (13)	0.0358 (3)	
C11	0.19816 (19)	1.10289 (14)	0.44465 (16)	0.0441 (3)	
H11	0.1640	1.1909	0.3742	0.053*	
C12	0.15988 (19)	1.09807 (16)	0.57670 (17)	0.0469 (3)	
H12	0.0981	1.1843	0.5963	0.056*	
C13	0.21185 (17)	0.96457 (16)	0.68549 (15)	0.0413 (3)	
C14	0.1701 (2)	0.9614 (2)	0.82289 (17)	0.0541 (4)	
H14	0.1102	1.0485	0.8415	0.065*	
C15	0.2156 (2)	0.8347 (2)	0.92698 (17)	0.0596 (4)	
H15	0.1890	0.8359	1.0161	0.071*	
C16	0.3028 (2)	0.7010 (2)	0.90168 (15)	0.0545 (4)	
H16	0.3306	0.6140	0.9742	0.065*	
C17	0.34726 (19)	0.69747 (16)	0.77121 (13)	0.0422 (3)	
H17	0.4051	0.6082	0.7558	0.051*	
C18	0.30558 (16)	0.82910 (14)	0.65996 (12)	0.0344 (2)	
C19	0.34334 (15)	0.83897 (12)	0.52100 (12)	0.0310 (2)	
O1	0.67384 (13)	0.57343 (10)	0.60386 (9)	0.0374 (2)	
O2	0.24447 (12)	0.73626 (10)	0.28658 (9)	0.0376 (2)	
H2	0.268 (3)	0.630 (2)	0.317 (2)	0.066 (6)*	
O3	0.32112 (19)	1.03639 (12)	0.17284 (11)	0.0592 (3)	
N1	0.43075 (13)	0.72309 (10)	0.46469 (9)	0.0303 (2)	

### Atomic displacement parameters (Å^2^)

**Table:**

	*U*^11^	*U*^22^	*U*^33^	*U*^12^	*U*^13^	*U*^23^
C1	0.0310 (5)	0.0265 (5)	0.0314 (5)	−0.0044 (4)	−0.0037 (4)	−0.0091 (4)
C2	0.0318 (5)	0.0332 (5)	0.0334 (5)	−0.0057 (4)	−0.0021 (4)	−0.0128 (4)
C3	0.0329 (6)	0.0468 (7)	0.0462 (7)	−0.0022 (5)	−0.0040 (5)	−0.0221 (6)
C4	0.0366 (6)	0.0628 (9)	0.0545 (8)	−0.0073 (6)	0.0071 (6)	−0.0317 (7)
C5	0.0490 (8)	0.0656 (9)	0.0402 (7)	−0.0176 (7)	0.0120 (6)	−0.0237 (7)
C6	0.0460 (7)	0.0487 (7)	0.0309 (6)	−0.0114 (5)	0.0016 (5)	−0.0106 (5)
C7	0.0339 (5)	0.0342 (5)	0.0308 (5)	−0.0075 (4)	−0.0004 (4)	−0.0096 (4)
C8	0.0328 (5)	0.0321 (5)	0.0268 (5)	−0.0041 (4)	−0.0038 (4)	−0.0057 (4)
C9	0.0367 (6)	0.0319 (5)	0.0370 (6)	−0.0045 (4)	−0.0026 (4)	−0.0040 (4)
C10	0.0323 (5)	0.0302 (5)	0.0430 (6)	−0.0053 (4)	−0.0014 (4)	−0.0104 (5)
C11	0.0384 (6)	0.0293 (6)	0.0628 (8)	−0.0041 (5)	−0.0020 (6)	−0.0146 (5)
C12	0.0378 (6)	0.0398 (7)	0.0697 (9)	−0.0068 (5)	0.0044 (6)	−0.0297 (7)
C13	0.0310 (5)	0.0490 (7)	0.0540 (7)	−0.0105 (5)	0.0038 (5)	−0.0301 (6)
C14	0.0438 (7)	0.0725 (10)	0.0611 (9)	−0.0126 (7)	0.0084 (6)	−0.0447 (9)
C15	0.0510 (8)	0.0929 (13)	0.0467 (8)	−0.0148 (8)	0.0065 (6)	−0.0413 (9)
C16	0.0540 (8)	0.0735 (10)	0.0354 (7)	−0.0126 (7)	0.0017 (6)	−0.0188 (7)
C17	0.0428 (7)	0.0495 (7)	0.0346 (6)	−0.0087 (5)	0.0009 (5)	−0.0158 (5)
C18	0.0290 (5)	0.0408 (6)	0.0379 (6)	−0.0088 (4)	0.0016 (4)	−0.0184 (5)
C19	0.0278 (5)	0.0304 (5)	0.0353 (5)	−0.0060 (4)	0.0001 (4)	−0.0119 (4)
O1	0.0403 (5)	0.0356 (4)	0.0330 (4)	−0.0006 (3)	−0.0097 (3)	−0.0091 (3)
O2	0.0338 (4)	0.0389 (5)	0.0380 (4)	−0.0049 (3)	−0.0085 (3)	−0.0089 (4)
O3	0.0753 (8)	0.0408 (6)	0.0421 (5)	−0.0017 (5)	−0.0040 (5)	0.0047 (4)
N1	0.0325 (5)	0.0292 (4)	0.0265 (4)	−0.0019 (3)	−0.0028 (3)	−0.0080 (3)

### Geometric parameters (Å, °)

**Table:**

C1—O1	1.2192 (14)	C10—C19	1.3826 (16)
C1—N1	1.3908 (14)	C10—C11	1.4075 (17)
C1—C2	1.4829 (16)	C11—C12	1.359 (2)
C2—C3	1.3855 (16)	C11—H11	0.9300
C2—C7	1.3858 (16)	C12—C13	1.421 (2)
C3—C4	1.387 (2)	C12—H12	0.9300
C3—H3	0.9300	C13—C14	1.420 (2)
C4—C5	1.393 (2)	C13—C18	1.4307 (17)
C4—H4	0.9300	C14—C15	1.349 (3)
C5—C6	1.387 (2)	C14—H14	0.9300
C5—H5	0.9300	C15—C16	1.404 (3)
C6—C7	1.3885 (17)	C15—H15	0.9300
C6—H6	0.9300	C16—C17	1.3704 (18)
C7—C8	1.4983 (16)	C16—H16	0.9300
C8—O2	1.4071 (14)	C17—C18	1.4148 (19)
C8—N1	1.4905 (14)	C17—H17	0.9300
C8—C9	1.5445 (16)	C18—C19	1.4177 (16)
C9—O3	1.2037 (16)	C19—N1	1.4278 (14)
C9—C10	1.4616 (18)	O2—H2	0.96 (2)
			
O1—C1—N1	125.83 (10)	C11—C10—C9	127.75 (12)
O1—C1—C2	127.52 (10)	C12—C11—C10	118.08 (13)
N1—C1—C2	106.62 (9)	C12—C11—H11	121.0
C3—C2—C7	121.55 (11)	C10—C11—H11	121.0
C3—C2—C1	128.81 (11)	C11—C12—C13	121.64 (12)
C7—C2—C1	109.36 (10)	C11—C12—H12	119.2
C2—C3—C4	117.33 (12)	C13—C12—H12	119.2
C2—C3—H3	121.3	C14—C13—C12	120.91 (13)
C4—C3—H3	121.3	C14—C13—C18	118.03 (14)
C3—C4—C5	121.23 (13)	C12—C13—C18	121.06 (12)
C3—C4—H4	119.4	C15—C14—C13	121.45 (14)
C5—C4—H4	119.4	C15—C14—H14	119.3
C6—C5—C4	121.25 (12)	C13—C14—H14	119.3
C6—C5—H5	119.4	C14—C15—C16	120.45 (14)
C4—C5—H5	119.4	C14—C15—H15	119.8
C5—C6—C7	117.34 (13)	C16—C15—H15	119.8
C5—C6—H6	121.3	C17—C16—C15	120.69 (16)
C7—C6—H6	121.3	C17—C16—H16	119.7
C2—C7—C6	121.28 (11)	C15—C16—H16	119.7
C2—C7—C8	109.15 (10)	C16—C17—C18	120.21 (14)
C6—C7—C8	129.57 (11)	C16—C17—H17	119.9
O2—C8—N1	111.82 (9)	C18—C17—H17	119.9
O2—C8—C7	113.83 (9)	C17—C18—C19	125.32 (11)
N1—C8—C7	103.29 (9)	C17—C18—C13	119.13 (12)
O2—C8—C9	104.10 (9)	C19—C18—C13	115.52 (12)
N1—C8—C9	103.13 (9)	C10—C19—C18	121.92 (11)
C7—C8—C9	120.25 (10)	C10—C19—N1	109.84 (10)
O3—C9—C10	129.58 (12)	C18—C19—N1	128.21 (10)
O3—C9—C8	124.77 (12)	C8—O2—H2	107.6 (12)
C10—C9—C8	105.45 (9)	C1—N1—C19	126.89 (9)
C19—C10—C11	121.77 (12)	C1—N1—C8	111.06 (9)
C19—C10—C9	110.43 (10)	C19—N1—C8	108.74 (9)
			
O1—C1—C2—C3	1.8 (2)	C11—C12—C13—C18	−0.5 (2)
N1—C1—C2—C3	179.64 (12)	C12—C13—C14—C15	178.73 (14)
O1—C1—C2—C7	−172.16 (12)	C18—C13—C14—C15	−0.5 (2)
N1—C1—C2—C7	5.70 (13)	C13—C14—C15—C16	−1.3 (2)
C7—C2—C3—C4	1.3 (2)	C14—C15—C16—C17	1.6 (3)
C1—C2—C3—C4	−171.95 (12)	C15—C16—C17—C18	0.0 (2)
C2—C3—C4—C5	−0.3 (2)	C16—C17—C18—C19	−179.45 (13)
C3—C4—C5—C6	−1.2 (2)	C16—C17—C18—C13	−1.7 (2)
C4—C5—C6—C7	1.6 (2)	C14—C13—C18—C17	2.00 (18)
C3—C2—C7—C6	−0.90 (19)	C12—C13—C18—C17	−177.24 (12)
C1—C2—C7—C6	173.56 (11)	C14—C13—C18—C19	179.92 (11)
C3—C2—C7—C8	177.95 (11)	C12—C13—C18—C19	0.68 (17)
C1—C2—C7—C8	−7.60 (13)	C11—C10—C19—C18	0.37 (18)
C5—C6—C7—C2	−0.6 (2)	C9—C10—C19—C18	−177.05 (10)
C5—C6—C7—C8	−179.17 (13)	C11—C10—C19—N1	178.75 (11)
C2—C7—C8—O2	−115.06 (11)	C9—C10—C19—N1	1.33 (14)
C6—C7—C8—O2	63.66 (17)	C17—C18—C19—C10	177.17 (12)
C2—C7—C8—N1	6.39 (12)	C13—C18—C19—C10	−0.61 (17)
C6—C7—C8—N1	−174.89 (13)	C17—C18—C19—N1	−0.9 (2)
C2—C7—C8—C9	120.48 (11)	C13—C18—C19—N1	−178.66 (10)
C6—C7—C8—C9	−60.81 (18)	O1—C1—N1—C19	40.41 (18)
O2—C8—C9—O3	−72.49 (16)	C2—C1—N1—C19	−137.50 (11)
N1—C8—C9—O3	170.63 (13)	O1—C1—N1—C8	176.42 (11)
C7—C8—C9—O3	56.47 (18)	C2—C1—N1—C8	−1.48 (12)
O2—C8—C9—C10	102.75 (10)	C10—C19—N1—C1	125.80 (12)
N1—C8—C9—C10	−14.13 (12)	C18—C19—N1—C1	−55.95 (17)
C7—C8—C9—C10	−128.29 (11)	C10—C19—N1—C8	−11.01 (13)
O3—C9—C10—C19	−176.69 (14)	C18—C19—N1—C8	167.23 (11)
C8—C9—C10—C19	8.38 (13)	O2—C8—N1—C1	119.96 (10)
O3—C9—C10—C11	6.1 (2)	C7—C8—N1—C1	−2.84 (12)
C8—C9—C10—C11	−168.84 (12)	C9—C8—N1—C1	−128.76 (10)
C19—C10—C11—C12	−0.18 (19)	O2—C8—N1—C19	−95.95 (11)
C9—C10—C11—C12	176.77 (12)	C7—C8—N1—C19	141.25 (9)
C10—C11—C12—C13	0.3 (2)	C9—C8—N1—C19	15.33 (11)
C11—C12—C13—C14	−179.76 (13)		

### Hydrogen-bond geometry (Å, °)

**Table:**

*D*—H···*A*	*D*—H	H···*A*	*D*···*A*	*D*—H···*A*
O2—H2···O1^i^	0.96 (2)	1.85 (2)	2.7978 (15)	172.2 (17)
C6—H6···O3^ii^	0.93	2.52	3.1609 (18)	126

Symmetry codes: (i) −*x*+1, −*y*+1, −*z*+1; (ii) −*x*+1, −*y*+2, −*z*.
